# Vegetable Extracts and Nutrients Useful in the Recovery from *Helicobacter pylori* Infection: A Systematic Review on Clinical Trials

**DOI:** 10.3390/molecules26082272

**Published:** 2021-04-14

**Authors:** Hammad Ullah, Alessandro Di Minno, Cristina Santarcangelo, Haroon Khan, Jianbo Xiao, Carla Renata Arciola, Maria Daglia

**Affiliations:** 1 Department of Pharmacy, University of Naples Federico II, 80131 Naples, Italy; hammad.ullah@unina.it (H.U.); alessandro.diminno@unina.it (A.D.M.); cristina.santarcangelo@unina.it (C.S.); 2 CEINGE-Biotecnologie Avanzate, 80131 Naples, Italy; 3 Department of Pharmacy, Abdul Wali Khan University, Mardan 23200, Pakistan; hkdr2006@gmail.com or; 4 International Research Center for Food Nutrition and Safety, Jiangsu University, Zhenjiang 212013, China; jianboxiao@yahoo.com; 5 Department of Experimental, Diagnostic and Specially Medicine, University of Bologna, via San Giacomo 14, 40126 Bologna, Italy; carlarenata.arciola@unibo.it; 6 Laboratorio di Patologia delle Infezioni Associate all’Impianto, IRCCS Istituto Ortopedico Rizzoli, 40136 Bologna, Italy

**Keywords:** *Helicobacter pylori*, alternative therapies, vegetable extracts, micronutrients, clinical trials

## Abstract

*Helicobacter pylori* (*H. pylori*) infections affect almost half of the world’s population, with gradually increasing incidence in developed countries. Eradication of *H. pylori* may provide significant benefits to the affected individual by healing a number of gastrointestinal and extra-digestive disorders. But due to increased microbial resistance and lack of patient adherence to the therapy, the eradication rate of *H. pylori* is below 80% with current pharmacological therapies. The usage of botanicals for their therapeutic purposes and medicinal properties have been increased in last decades. They can be use as alternative *H. pylori* treatments, especially against drug-resistant strains. Epidemiological studies have revealed that people with lower vegetable and micronutrient intake may be at increased risk of *H. pylori* infection. We have undertaken a review of clinical trials to evaluate the efficacy of vegetable extracts and micronutrients in patients with *H. pylori*. Various databases, such as Google Scholar, PubMed, Scopus, Web of Science, and the Cochrane Library, were searched for the articles published in English. A total of 24 clinical studies (15 for vegetable extracts and 9 for micronutrients) were selected to be reviewed and summarized in this article. Vegetable extracts (Broccoli sprouts, curcumin, Burdock complex, and *Nigella sativa*) and micronutrients (vitamin C and E) were not found to be as effective as single agents in *H. pylori* eradication, rather their efficacy synergized with conventional pharmacological therapies. Conversely, GutGard was found to be significantly effective as a single agent when compared to placebo control.

## 1. Introduction

*Helicobacter pylori* is a Gram-negative, spiral shaped microaerophilic bacterium, infecting half of the world population. However, the incidence of infection has been gradually decreasing in developed countries due to reduced family sizes, decreased overcrowding, and improved sanitization. The prevalence in developing countries is around 90%, while in developed countries (except Japan), the prevalence of infection falls below 40% [1,2]. *H. pylori* is a nasty pathogen that can persist in the stomach of infected persons for a lifetime, if left untreated. It provokes a chronic gastric inflammatory response, resulting in the development of several gastric pathological conditions including superficial gastritis, chronic atrophic gastritis, peptic ulcers, gastric cancer, and mucosa-associated lymphoid tissue (MALT) lymphoma [3]. In 1994, it was regarded as a primary factor for the development of gastric cancer and was classified as a group-I carcinogen by The International Agency for Research on Cancer [4] *H. pylori* bacteria have also been identified in extra-gastric tissues in the head and neck regions, with unknown origin and pathogenicity [5]. The possible mechanisms of diffusions are gastric reflux and nasal or oral routes, where laryngopharyngeal reflux may contribute to many sinonasal, laryngeal, pharyngeal, and middle ear disorders; however, there is no clear evidence of the active role of *H. pylori* in otorhinolaryngological diseases [5]. *H. pylori* infection has also been linked with some extra-digestive diseases i.e., iron deficiency anemia [6], idiopathic thrombocytopenic purpura [7,8], hepatobiliary diseases [9,10], nonalcoholic fatty liver disease [11], diabetes mellitus [12,13], cardiovascular disorders [14,15], neurodegeneration (Alzheimer’s disease, Parkinson’s disease, and glaucoma) [16,17,18], and osteoporosis [19,20,21]. Osteoporosis, with the burden of bone fragility and osteoporotic fractures it brings, is a common multifactorial disorder of increasing incidence. A large meta-analysis demonstrated that patients with *H. pylori* infection are at a high risk of developing osteoporosis [21]. Interestingly, recent data suggests that osteoporosis and many of the extra-digestive diseases associated with *H. pylori* infection share risk factors and pathogenetic pathways [19]. In this connection, it can be noted that diabetes mellitus, a prominent extra-digestive disease associated to *H. pylori* infection, adversely impacts on skeleton and bone health, and is associated with an increased risk of osteoporosis and fragility fractures [22]. Infection with the most virulent strains (in particular, cagA+) appears to be associated with higher inflammatory response and elevated risk of gastroduodenal and extra-digestive diseases. Though details still remain unclear, person-to-person transmission and familial spread are the most common routes of the infection transmission [23,24].

Eradication of *H. pylori* may provide significant benefits to the affected individual in term of the healing of a number of gastrointestinal complications and extra-gastrointestinal disorders [25]. The current pharmacological therapy is based on the combination of antimicrobial and antisecretory agents, as an increase in gastric pH by antisecretory agents is required for the bactericidal action of antibiotics. Many antibacterial entities are currently in practice for the treatment of *H. pylori* infections such as amoxicillin, tetracycline, clarithromycin, metronidazole, levofloxacin, bismuth subsalicylate, and rifabutin, while the only antisecretory class in use is proton pump inhibitors (PPIs) [26]. Different guidelines are available for treatments, where prescribers rely on triple therapy (amoxicillin, clarithromycin, and PPI) for 7–14 days as a first line therapy in most cases [27,28,29]. The eradication rate remains below 80% with this regimen, because of increased microbial resistance to clarithromycin, and switching to a quadruple therapy by the addition of bismuth-containing compounds has been suggested [30,31]. In addition to microbial resistance, lack of patient adherence to the therapy is an important factor responsible for treatment failure. The main cause of patient nonadherence is the complexity of the therapy involving at least three drugs, administered in frequent doses and for a long time. Other causes of nonadherence may include adverse drug reactions to the therapy with lack of immediate improvement, high cost of the medications, and recurrence of the infection after a successful eradication [3].

Considering these problems, novel therapeutic approaches, and the discovery of new molecules with antibacterial effects or adjuvants that may help patients to comply with the therapies have emerged. Natural extracts, especially those derived from botanical sources, have been used for their beneficial health effects and for the management of infectious disorders since ancient times [32]. A number of scientific studies have been reported the anti-*H. pylori* effects of natural products using in vitro and in vivo experimental models, either in the form of botanical extracts or pure compounds [33]. In this review, we focus on evaluating the efficacy of vegetable extracts and micronutrients against *H. pylori* by summarizing the most relevant clinical studies.

## 2. Methodology

**Figure 1:**
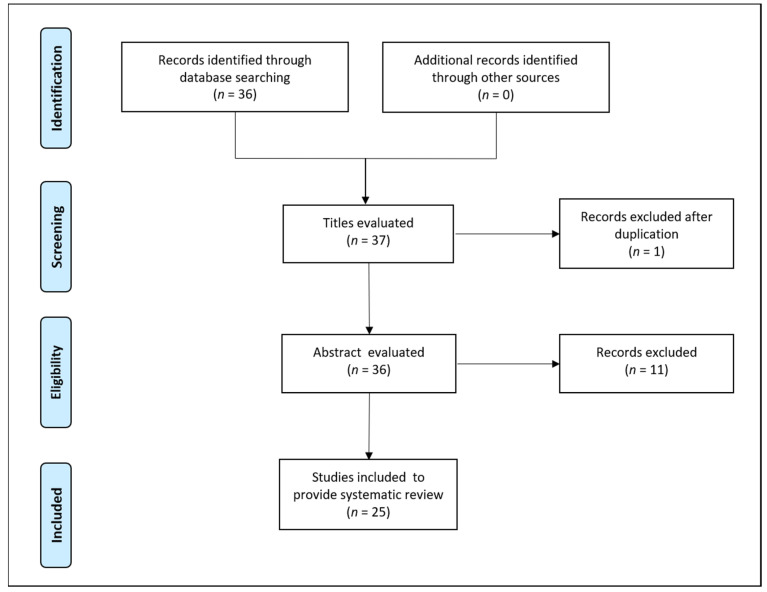
PRISMA flow diagram, showing the process of study selection.

The present study consists of an up-to-date review of clinical trials covering the importance of vegetable extracts and micronutrients supplementation, with regards to the eradication of *H. pylori* infections. The search involved all the clinical trials (randomized and non-randomized) involving the evaluation of vegetable extracts and micronutrients in patients with *H. pylori* and related gastrointestinal symptoms. We systematically searched databases including PubMed, Scopus, Web of Science and Cochrane Library for articles published in English language. The following terms were used in the literature search in all possible combinations: “*Helicobacter pylori* infection” and “vegetable extracts” or “cinnamon” or “broccoli sprouts” or “turmeric” or “curcumin” or “garlic” or “*Sesamum indicum*” or “*Glycyrrhiza glabra*” or “*Nigella sativa*” or “micronutrients” or “vitamins” or “vitamin C” or “vitamin E” or “minerals” or “zinc” or “polaprezinc” and “clinical trials”. At first, two independent researchers performed the selection of studies, and the third investigator was involved to carefully reviewed the studies selected, with duplicated or non-relevant studies being excluded after screening of title and abstracts. No limits were defined in the selection of clinical studies regarding randomization or nonrandomization and patient sex or age. All studies reporting in English language were selected for evaluation in writing this systematic review Overall, by systematic search of the databases, 37 potentially eligible studies were identified based on the inclusion criteria. After excluding duplicates and other articles due to valid reasons, we selected 25 articles to provide a systematic review. Figure 1 illustrates the PRISMA flow diagram for study selection.

## 3. Preclinical Studies on *H. pylori* Infections

Plants and spices have gained increased popularity for therapeutic purposes owing to their medicinal properties, in combination with their broad flexibility and favorable safety profiles, and they can be used as alternative anti-*H. pylori* formulations especially against drug-resistant strains [34]. There are hundreds of scientific publications describing the active antibacterial role of herbal products and nutrients against *H. pylori* [3]. Epidemiological studies have revealed that people with lower vegetable and micronutrients intake may be at increased risk of *H. pylori* infections [35,36]. Vegetable extracts can act against *H. pylori* infection via multiple mechanisms such as antibacterial, anti-adhesive and anti-inflammatory activities [37]. *Pisum Sativum* L. germinated in the dark have been showed to inhibit *H. pylori* growth in vitro in a dose dependent manner [38]. *Allium tuberosum* Rottler ex Spreng. extract was found to inhibit all 21 of the tested strains of *H. pylori* in vitro, with growth inhibitory zones ranging from 12 to 29 mm [39]. Another study showed the bactericidal effects of olive oil polyphenolic compounds against eight strains of *H. pylori* in vitro, and most importantly three of these were resistant to certain antibiotics [40]. These polyphenols could diffuse from oil into gastric juice and can be stable for hours in acidic environments.

Garlic (*Allium sativum* L.) oil significantly suppressed the *H. pylori* viability in vitro at concentrations of 2–32 mg in postprandial gastric volumes of 0.25–1 L [41]. In another study, garlic extracts suppressed the early stages of *H. pylori* induced gastritis in Mongolian gerbils when administered four hours following *H. pylori* inoculation at 1, 2, and 4% concentrations [42]. Isothiocyanate sulphoraphane (an abundant compound in broccoli sprouts) has been reported as a potent bacteriostatic agent against bacterium *H. pylori* (in vitro tested against 48 strains). It was effective in eradicating *H. pylori* from human gastric xenografts in nude mice [43]. An in vivo approach of evaluating the efficacy of fresh broccoli sprouts demonstrated that oral treatment of C57BL mice with sulphoraphane rich broccoli sprouts resulted in the reduction of *H. pylori* colonization, mucosal expression of TNF-α and IL-1β and alleviated corpus inflammation [44]. Anti-adhesive effects have been found with *Abelmoschus esculentus* (L.) Moench (okra) on *H. pylori* by Messing et al. using human gastric adenocarcinoma cell-lines in a dose dependent fashion (0.2–2 mg/mL) [45]. The analysis of the structures of polysaccharides from immature okra have confirmed the presence of acetylated rhamnogalacturonan-I polymers, decorated with short galactose side chains, possessing anti-adhesive potential [46]. 

*Glycyrrhiza glabra* L. (liquorice) has proven useful in the treatment of peptic ulcers in traditional therapeutic systems such as Chinese, Kampo and Indian medicine, supported by numerous studies evaluating the in vitro anti-*H. pylori* effects of liquorice [47,48,49]. A flavonoid rich extract of *G. glabra* has been found to be active against *H. pylori* with minimal inhibitory concentration (MIC) values of 32–64 μg/mL, with glabridin (the major flavonoid present in the extract) being the most potent antibacterial compound [50]. The anti-*H. pylori* mechanisms reported were inhibition of DNA gyrase, dihydrofolate reductase or protein synthesis. Moreover, no significant effects were found on the adhesion of *H. pylori* bacterium to the human gastric adenocarcinoma cell-line. The in vivo model revealed a reduction of *H. pylori* colonization in C57BL mice, when treated with *G. glabra* extract at a dose of 25 mg/kg for 3 weeks [51]. Dietary supplementation of mice with *Angelica keiskei* inhibited *H. pylori* induced gastric inflammation, perhaps due to its antioxidant activity [52]. It was shown to prevent the increase of *H. pylori* induced lipid peroxide and myeloperoxidase activity, inhibited the neutrophils infiltration, and downregulated the expression of inflammatory mediators including interferon gamma (IFN-γ), inducible nitric oxide synthase (iNOS), and cyclooxygenase-2 (COX-2). The mastic gum derived from *Pistacia lentiscus* L. was reported to eliminate *H. pylori* pathogens in 1998 [53]. The essential oil isolated from *P. lentiscus* leaves was found to be effective against all *H. pylori* clinical isolates including drug resistant strains [54]. The reduction of *H. pylori* colonization with mastic gum extract was supported by in vivo study conducted by Paraschos and colleagues; however, attenuation of *H. pylori*-associated chronic inflammation was not observed with the treatment [55]. 

DNA damaging free radicals are usual products of chronic *H. pylori* infections, and supplementation with antioxidants such as vitamins (C and E) and carotenoid (astaxanthin) may be a useful strategy in combating *H. pylori* pathogenesis, with fruits and vegetables being the main dietary sources of these antioxidants [56]. Most of studies have been performed with vitamin C, which is known to highly concentrate in gastric mucosa and gastric juice, and which may influence the course of *H. pylori* infections and lower the risk of gastric cancer through several mechanisms [57,58]. It positively regulates the stimulation and activity of granulocytes, macrophages, and lymphocytes, and the production of immunoglobulin [36]. Vitamin C at high doses has been shown to inhibit the colonization of *H. pylori* in the stomach of Mongolian gerbils [59]. Wang et al. reported the in vitro inhibition of *H. pylori* growth and in vivo decrease in colonization levels and inflammation scores in mice treated with vitamin C and astaxanthin [60]. Treatment with astaxanthin may alters immune response to *H. pylori* by shifting Th1 response towards Th2 cell-response [61]. 

## 4. Vegetable Extracts and *H. pylori* Infections: Clinical Studies

A total of 16 studies reported the bioactivity of the vegetable extracts in patients with *H. pylori* infections and related symptoms or conditions (Table 1). Nir et al. conducted a randomized control trial (RCT) to evaluate the effects of cinnamon extract against *H. pylori* infection in human subjects who underwent gastroscopy [62]. Fifteen patients (4 males and 11 females) aged 16–79 years were treated with alcoholic cinnamon extract (40 mg twice daily) for 4 weeks, while eight patients (1 male and 7 females) aged 35–79 years were given placebo. Twenty-three patients completed the trial, and seven were excluded from the final analysis for one of the following reasons: (1) Negligible count on urea breath test despite of the presence of bacteria, (2) noncompliance to the therapy, and (3) receiving antibiotics during the study period. The results were evaluated by a ^13^C urea breath test, which showed a slight improvement in urea breath counts; however, no significant difference was found between groups before and after treatment.

A comparative study evaluated the potential of broccoli sprouts powder and standard triple therapy to eradicate *H. pylori* in patients with type 2 diabetes, either administered alone or in combination [64]. Eighty-six diabetic patients with positive *H. pylori* stool antigen test were randomized to receive broccoli sprout powder (6 g/day) for 4 weeks, standard triple therapy (omeprazole 20 mg, clarithromycin 500 mg, amoxicillin 1000 mg, twice a day) for 14 days, or a combination of broccoli sprout powder and standard triple therapy. At the end of the treatment period, *H. pylori* eradication was assessed by an *H. pylori* stool antigen test, which showed *H. pylori* eradication rates of 56%, 89.3%, and 91.7% with broccoli sprout powder, standard triple therapy, and combination of both, respectively. In addition, broccoli sprout powder also improved cardiovascular risk factors.

A RCT by Chang and colleagues did not show any improvement in *H. pylori* density with *Brassica oleracea* L. (broccoli sprout) extract but it inhibited the lipid peroxidation in gastric mucosa and thus could be beneficial for cytoprotection in *H. pylori* induced gastritis [65]. Volunteer subjects (*n* = 100) were evaluated, and eligible candidates were randomly assigned into three groups i.e., group A (*n* = 33) including *H. pylori* positive, broccoli sprout extract containing sulforaphane treatment subjects, group B (*n* = 28) including placebo subjects, and group C (*n* = 28) including *H. pylori* negative, broccoli sprout extract containing sulforaphane treatment patients. Patients were treated either with placebo or broccoli sprout capsules containing 250-mg standardized broccoli sprout yielding 1000 mg sulforaphane twice daily for 4 weeks. Result analysis revealed that urea breath test values or ammonia concentration were not significantly affected by treatment with broccoli sprout extract in *H. pylori* positive patients; however, malondialdehyde (MDA) values were significantly reduced in the intervention groups (groups A and C).

Two clinical studies showed an improvement of dyspeptic symptoms with curcumin in *H. pylori* positive patients, with no effects on *H. pylori* eradication. Twenty-five *H. pylori* positive patients including both males and females (mean age of 50 ± 12 years) with functional dyspepsia were treated with curcumin 30 mg, bovine lactoferrin 100 mg, N-acetylcysteine 600 mg, and pantoprazole 20 mg twice daily for 7 days [66]. Results revealed a significant improvement in the severity of symptoms and serologic signs of gastric inflammation in all patients, but *H. pylori* was eradicated in only three patients (the eradication rate was 12%). In a randomized double-blind placebo-controlled parallel-group trial, patients with peptic ulcer were assigned a standard triple therapy (clarithromycin 500 mg, amoxicillin 1000 mg, and pantoprazole 40 mg twice daily), and were randomized to receive either curcumin (500 mg/day) or placebo as adjuncts to standard triple therapy [67]. Adjunctive therapy with curcumin resulted in a greater improvement of dyspeptic symptoms as measured by Hong Kong dyspepsia index (HKDI) scores, however no significant effects were observed on *H. pylori* eradication with curcumin adjunction.

Judaki et al. observed an amelioration of oxidative stress and histopathologic changes with curcumin in combination with triple therapy in chronic gastritis associated with *H. pylori* infection [68]. Patients were randomized to receive triple therapy or curcumin in combination with triple therapy, 50 patients each. Treatment with triple therapy included omeprazole, amoxicillin, and metronidazole twice a day for one week, where turmeric tablets were administered at a dosage of 700 mg thrice a day for 28 days. Triple therapy in combination with curcumin significantly reduced MDA concentrations with an increase in total antioxidant capacity, as compared to patients treated with triple therapy alone.

Garlic consumption showed an eradication of *H. pylori* in RCT, though the results were not considerable when compared with placebo group [69]. Thirty-six patients (47% males and 53% females), with mean ages of 40.87 ± 16.45 years in the treatment group and 35.40 ± 11.26 years in the control group, were randomized to receive either two tablets of garlic powder daily (2 g each) or 2 tablets of placebo for 8 weeks. At the end of the experimental period, urea breath tests showed 87% *H. pylori* negative cases in the treatment group and 73% *H. pylori* negative cases in the control group. A pilot study conducted earlier, demonstrated non-satisfactory results with the intake of gastric oil capsules on *H. pylori* eradication [70]. Twenty dyspeptic patients aged 18–75 years with positive *H. pylori* were assigned to receive garlic oil capsules (4 mg) four times daily with meals for 14 days. Five patients completed the study, and no evidence of symptomatic improvement or *H. pylori* eradication was noted with garlic oil.

Graham and Lang performed a prospective crossover study in healthy *H. pylori* infected adults (average age: 41.4 years) to investigate the *H. pylori* treatment potential of garlic and jalapeño peppers [71]. Twelve subjects were participated in the study and were received garlic (10 freshly sliced cloves), capsaicin (six freshly sliced large jalapeño peppers) and two tablets of bismuth subsalicylate, via test meals on separate days. Neither garlic nor capsaicin possessed any beneficial effects on *H. pylori* eradication, as the median urease activity for garlic was 28.5 before and 39.8 after, while for capsaicin it was 43.7 before and 46.6 after. Bismuth showed a marked inhibitory potential with 55.8 vs. 14.3 median urease activity before and after therapy. A randomized, double-blind placebo-controlled clinical trial exhibited a positive response with Burdock complex (comprised of *Arctium lappa* L., *Angelica sinensis* (Oliv.) Diels, *Lithospermum erythrorhizon* Siebold & Zucc., and *Sesamum indicum* L. oil) against *H. pylori* [72]. Forty volunteers were randomly assigned to Burdock complex or placebo (*n* = 20 each), and they were directed to consume two bottles of Burdock complex or placebo (2 × 10 mL) every day after breakfast and dinner for eight weeks. Result analysis revealed a significant decrease in urea breath counts and inflammatory markers (TNF-α and IL-8) with improved antioxidant capacity (total phenolic contents, superoxide dismutase, and catalase).

GutGard (Root extract of *Glycyrrhiza glabra* L.) demonstrated considerable efficacy against *H. pylori* in a randomized, double-blind placebo-controlled study [73]. Participants diagnosed with *H. pylori* were assigned to receive GutGard 150 mg (*n* = 55) or placebo (*n* = 52) daily for 60 days. At the end of experiment, *H. pylori* stool antigen (HpSA) test was found negative in about 56% of patients treated with GutGard while HpSA was negative in only 4% of patients in placebo group. Salem and colleagues conducted a comparative study of *Nigella sativa* and standard triple therapy in eradication of *H. pylori* in patients (*n* = 88; 32 males and 56 females; age range of 18–65 years) with non-ulcer dyspepsia [74]. Patients were randomized to four groups i.e., triple therapy (clarithromycin, amoxicillin, and omeprazole), 1-g *N. sativa* + 40 mg omeprazole, 2-g *N. sativa* + 40 mg omeprazole, and 3-g *N. sativa* + 40 mg omeprazole. The difference in *H. pylori* eradication rate was not significant between triple therapy (82.6%) and 2-g *N. sativa* (66.7%), while the eradication rates demonstrated by 1- and 3-g *N. sativa* were less significant. However, the improvement of dyspeptic symptoms was similar in all groups.

The efficacy of *G. glabra* has also been evaluated in patients with *H. pylori* synergistically with a probiotic strain *Lactobacillus paracasei*, in randomized, double-blind, placebo-controlled trials [75]. A total of 142 patients were randomly allocated to the treatment group (fermented milk containing 1.0 × 10^6^ CFU/mL *L. paracasei* HP7 and 100 mg *G. glabra*) or placebo group (fermented milk only) once daily for 8 weeks. A significant improvement in gastrointestinal symptoms, ^13^C-urea breath test scores, and chronic inflammation was observed in the treatment group. Addition of *G. glabra* to a clarithromycin-based regimen showed increased efficacy in *H. pylori* eradication [76]. In RCT, 120 patients affected by non-ulcer dyspepsia or peptic ulcer disease were randomized into a treatment group (clarithromycin based triple regimen + *G. glabra* 380 mg twice daily) or a control group (clarithromycin based triple regimen) for 2 weeks. *H. pylori* eradication rate for treatment and control groups was found to be as 83.3% and 62.5%, respectively.

A double-blind, randomized controlled clinical trial demonstrated significant improvement in patients with functional dyspepsia with adjuvant supplementation of honey-based *N. sativa* formulation [77]. Patients (*n* = 70) with functional dyspepsia according to ROME III criteria, confirmed by upper gastrointestinal endoscopy, were allocated a treatment of a combination of anti-secretory agent and honey-based formulation of *N. sativa* (5 mL *N. sativa*) once daily or placebo for 8 weeks. The mean Hong Kong index of dyspepsia scores and *H. pylori* infection rates were significantly lower in the *N. sativa* treated group, with no serious adverse event being reported.

Mastic gum demonstrated nonsignificant effects on *H. pylori* eradication in a randomized pilot study [77], however the antimicrobial effects were consistent in preclinical studies. Fifty-two patients were randomized in four groups to receive either pure mastic gum (350 mg, three times daily) for 14 days (group A), pure mastic gum (1 g, three times daily) for 14 days (group B), combination of pure mastic gum (350 mg three times daily) and pantoprazole (20 mg twice daily) for 14 days (group C) or standard therapy (pantoprazole 20 mg, amoxicillin 1 g and clarithromycin 500 mg) for 10 days (group D). *H. pylori* eradication was confirmed with urea breath test 5 weeks after the completion of eradication therapy, which revealed a *H. pylori* eradication in 4/13 patients in group A and in 5/13 patients in group B, while none of the patients treated showed eradication in group C. Briefly, there were no significant differences in the mean values of urea breath test in these three groups. On the other hand, the patients treated with standard therapy appeared to have significant eradication of the infection, i.e., 10/13 patients with negative urea breath test [78]. 

## 5. Micronutrients and *H. pylori* Infections: Clinical Studies

Overall, the 9 human studies included in this review have demonstrated the efficacy of nutrients against *H. pylori* infections (Table 2). RCT conducted by Zojaji et al. revealed a significant increase in *H. pylori* eradication rate with the augmentation of standard therapy with vitamin C [79]. Patients were randomized into group A (*n* = 162; mean age: 45 years) receiving standard therapy (amoxicillin 1 g, metronidazole 500 mg, bismuth 240 mg and omeprazole 40 mg) and group B (*n* = 150; mean age: 43 years) receiving the same regimen plus vitamin C 500 mg/day, for two weeks. The *H. pylori* eradication rates in group A and group B were 48.8% and 78% respectively, showing negative urea breath tests. Sezikli et al. observed the improved eradication rates of *H. pylori* by the co-supplementation of triple therapy with vitamins C and E [80]. Two hundred patients were randomized to receive either standard triple therapy (lansoprazole, amoxicillin, and clarithromycin) for 14 days plus vitamins C (500 mg) and E (200 mg) for 30 days or standard triple therapy alone for 14 days. Urea breath test showed significantly higher eradication rates in patients receiving vitamins C and E.

Another trial conducted by Sezikli et al. investigated the synergistic effects of vitamin C and vitamin E in *H. pylori* eradication rates when co-supplemented with triple therapy, and their possible link with oxidative stress alteration [81]. The study included 160 patients, randomized to receive either conventional anti-*H. pylori* therapy (lansoprazole, amoxicillin, clarithromycin, and bismuth subcitrate) for 2 weeks plus vitamins C (1000 mg/day) and E (400 IU/day) for one month, or conventional therapy alone for 2 weeks. *H. pylori* eradication rates were significantly higher in patients receiving vitamins C and E; however, no difference was found in total antioxidant capacity between both groups. Supplementation with antioxidant vitamins (C and E) possessed no significant effects on mucosal reactive oxygen species damage in *H. pylori* induced gastritis [82]. *H. pylori* positive patients (*n* = 117) were randomized into four groups to receive triple therapy alone (Bismuth chelate, tetracycline, and metronidazole for 2 weeks), vitamins C (200 mg) and E (50 mg) twice a day for 4 weeks, combination of both treatments or placebo MDA levels and reactive oxygen species were reduced in *H. pylori* eradicated patients, but no considerable effects were found with vitamin supplementation.

The RCT conducted by Chuang and colleagues revealed that an add on treatment of standard *H. pylori* therapy with vitamin C can reduce the daily dosage of clarithromycin while preserving the high eradication efficacy for clarithromycin susceptible *H. pylori* patients [83]. *H. pylori* infected patients (*n* = 171) were assigned to one-week triple therapies, including omeprazole and amoxicillin plus either one of the following twice daily: (1) Clarithromycin 250 mg; (2) clarithromycin 250 mg and vitamin C 500 mg; (3) clarithromycin 500 mg. The patients receiving a combination of clarithromycin 250 mg and vitamin C showed a higher eradication rate than clarithromycin 250 mg alone, where the equivalent rate of this combination was equivalent to clarithromycin 500 mg.

Conversely a study evaluating the effects of adjunctive supplementation of triple and quadruple therapies with vitamins C and E on the eradication rates of *H. pylori*, showed no benefits of such supplementation [84]. Patients were divided in four groups i.e., one receiving triple therapy (amoxicillin, clarithromycin, and lansoprazole) for 2 weeks, a second was assigned to receive triple therapy for 2 weeks plus vitamin C (500 mg/day) and E (100 U/day) for one month, a third receiving quadruple therapy (amoxicillin, clarithromycin, lansoprazole, and bismuth subcitrate) for 2 weeks, and patients in group four were randomized to receive quadruple therapy for 2 weeks plus vitamins C and E for one month. *H. pylori* eradication rates were accessed using a C14 urea breath test, showing no considerable difference in *H. pylori* eradication rates between groups A and B, and similarly groups C and D.

The efficacy of polaprezinc (chelated form of zinc and L-carnosine) was tested in a randomized, parallel-group, open-label, controlled, prospective multicenter study in combination with triple therapy for *H. pylori*, and was compared with the patients assigned to receive triple therapy (omeprazole, amoxicillin, and clarithromycin) alone [85]. The patients were randomized into three groups for 14-day treatment; (A) patients received polaprezinc 75 mg plus triple therapy twice daily, (B) patients received polaprezinc 150 mg plus triple therapy twice daily, and (C) patients received triple therapy alone. Patients in groups A and B showed significantly higher eradication rates compared to group C, whereas no significant difference was found among groups A and B. No serious events were reported with polaprezinc, but the adverse event rate for group B was higher than for group A.

Another clinical trial conducted by Kashimura et al. showed an improvement of infection eradication with lansoprazole, amoxycillin and clarithromycin, when combined with polaprezinc [86]. Patients (*n* = 66) suffering from dyspeptic symptoms with *H. pylori* infections were randomized to receive one of the two regimens for 7 days twice daily, i.e., lansoprazole, amoxycillin, and clarithromycin, or lansoprazole, amoxycillin, and clarithromycin plus polaprezinc. Supplementation of peptic ulcer patients with zinc sulfate 220 mg/day did not show any clinically relevant benefits, however patients with normal zinc levels were found to have better ulcer treatment [86]. Patients (*n* = 90) with active peptic ulcer disease were randomized into an intervention group receiving standard triple therapy plus zinc, and a control group receiving standard triple therapy plus placebo for 2 weeks.

## 6. Conclusions

This systematic review provides relevant information regarding the efficacy of vegetable extracts and micronutrients in patients with *H. pylori* in clinical settings. Human studies have shown that vegetable extracts and micronutrients are not effective as single agents in eradication of *H. pylori* infections, but they may synergize with conventional pharmacological therapies for improved efficacy when used in combination. Broccoli sprouts, curcumin, Burdock complex, and vitamins (C and E) were found to be effective when used in combination with standard triple therapies for *H. pylori*. *Nigella sativa* L. in combination with omeprazole showed similar results to standard triple therapy consisting of antibiotics and omeprazole. Conversely GutGard was found significantly effective as a single agent when compared to placebo control in both clinical trials. Polaprezinc showed good efficacy in combination with standard therapy, but zinc showed no synergistic benefits.

## Figures and Tables

**Table 1:** Summary of clinical trials of vegetable extracts in *Helicobacter pylori* infection.

Study Design	Study Sample	Experimental Intervention	Control Intervention	Main Outcomes	References
RCT	32 patients underwent gastroscopy (23 patients completed trial)	Cinnamon extract (40 mg twice daily) for 4 weeks	Placebo	Slight and non-significant improvement in urea breath counts.	[62]
RCT	86 diabetic patients with positive *H. pylori* stool antigen test (77 patients completed trial)	Broccoli sprouts powder (6 g/day) alone or in combination with standard triple therapy for 4 weeks	Standard triple therapy (omeprazole 20 mg, clarithromycin 500 mg, amoxicillin 1000 mg)	*H. pylori* eradication rates with Broccoli sprouts powder, standard triple therapy, and combination of both were 56%, 89.3% and 91.7%, respectively.	[63]
RCT	89 volunteer subjects randomized in group A (*H. pylori* positive, broccoli sprout extract containing sulforaphane), group B (placebo) and group C (*H. pylori* negative, broccoli sprout extract containing sulforaphane).	One capsule containing 250 mg broccoli sprout extract yielding 1000 μg sulforaphane.	Placebo	No significant effect was found in treatment group regards to *H. pylori* infection density. MDA concentration was significantly reduced in groups A and C with broccoli sprout treatment.	[64]
Controlled clinical trial	25 *H. pylori* positive patients (mean age: 50 ± 12 years) with functional dyspepsia	Curcumin 30 mg, bovine lactoferrin 100 mg, N-acetylcysteine 600 mg, and pantoprazole 20 mg twice daily for 7 days	-	Significant improvement of the severity of symptoms and serologic signs of gastric inflammation. 12% *H. pylori* eradication rate.	[65]
RCT	68 *H. pylori* positive patients aged 20–50 years with peptic ulcer (60 patients, 30 in each group completed study)	Curcumin 500 mg/day, as adjunct to standard triple therapy (clarithromycin 500 mg, amoxicillin 1000 mg, and pantoprazole 40 mg twice daily)	Placebo	Improvement of dyspepsia in curcumin group was significantly higher (27.6%) vs. placebo (6.7%). *H. pylori* eradication rate was 73.3% in both groups.	[66]
RCT	*H. pylori* patients were randomized in two groups, 50 patients each, with mean age of 54.65 ± 16.54 in triple therapy group, and 53.65 ± 15.65 in triple therapy + curcumin group.	Triple therapy twice a day for one week + turmeric tablets (700 mg) thrice a day for 28 days.	Triple therapy (omeprazole, amoxicillin, and metronidazole) twice a day for one week.	Significantly decrease in MDA levels and increase in TAC of the gastric mucosa in triple therapy + curcumin treated patients.	[67]
RCT	36 patients (47% males and 53% females) with mean age of 40.87 ± 16.45 years in the treatment group and 35.40 ± 11.26 years in the control group	2 tablets of garlic powder daily (2 g each) for 8 weeks.	Placebo	87% *H. pylori* negative cases in Garlic treated group and 73% *H. pylori* negative cases in placebo group, as confirmed by UBT.	[68]
Pilot study	20 dyspeptic patients aged 18–75 years with positive *H. pylori* (5 patients completed study)	Garlic oil capsule (4 mg) four times daily for 14 days	-	No improvement in *H. pylori* symptoms or eradication	[69]
Single-center, prospective crossover study	12 healthy *H. pylori* infected adults (average age: 41.4 years)	Garlic (10 freshly sliced cloves) with 3 meals per test day, Capsaicin (six freshly sliced large jalapeño peppers) with 3 meals per test day.	Bismuth subsalicylate (2 tablets) with 3 meals per test day	No beneficial effects by garlic or capsaicin on *H. pylori*. Median urease activity before and after therapy for garlic was 28.5 vs. 39.8, for capsaicin was 43.7 vs. 46.6, and for bismuth was 55.8 vs. 14.3.	[70]
RCT	40 volunteers with *H. pylori* infection	Burdock complex (*Arctium lappa*, *Angelica sinensis*, *Lithospermum erythrorhizon*, and *Sesamum indicum* oil) 2 bottles (2 × 10 mL) every day after breakfast and dinner, for 8 weeks.	Placebo	A significant decrease in urea breath counts and inflammatory markers (TNF-α and IL-8), and improved antioxidant capacity, with Burdock complex	[71]
RCT	107 participants with *H. pylori* infection, aged 18–45 years	GutGard (Root extract of *Glycyrrhiza glabra*) 150 mg for 60 days	Placebo	*H. pylori* stool antigen (HpSA) was negative in 56% and 4% of patients treated with GutGard or placebo, respectively	[72]
RCT	88 *H. pylori* patients, with non-ulcer dyspepsia (age range of 18–65 years)	1-, 2- or 3-g *Nigella sativa* + 40 mg omeprazole	Standard triple therapy (clarithromycin, amoxicillin, and omeprazole)	No significant difference in *H. pylori* eradication rate between triple therapy (82.6%) and 2-g *N. sativa* (66.7%). Effect on dyspeptic symptoms was similar in all groups.	[73]
RCT	142 patients aged 19–70 years were enrolled in the trial.	Fermented milk containing 1.0 × 10^6^ CFU/mL *L. paracasei* HP7 and 100 mg *G. glabra*.	Placebo	Significant improvement in GI symptoms, ^13^C-UBT scores, and chronic inflammation was observed in treatment group.	[74]
RCT	120 patients suffering from non-ulcer dyspepsia or peptic ulcer disease randomized into treatment group (mean age: 38.8 years) or control group (mean age: 40.1 years).	Clarithromycin based triple regimen + *G. glabra* 380 mg twice daily, for 2 weeks.	Clarithromycin based triple regimen (clarithromycin twice daily, amoxicillin once daily, omeprazole twice daily), for 2 weeks.	* H. pylori * eradication rate for treatment and control groups was found as 83.3% and 62.5%, respectively.	[75]
RCT	70 patients were randomized in treatment (mean age: 42.31713.85) and control (mean age: 36.31713.64) groups.	Anti-secretory agent ^1^ + honey-based formulation of *N. sativa* (5 mL *N. sativa*) once daily, for 8 weeks.	Placebo	The mean Hong Kong index of dyspepsia scores and *H. pylori* infection rates were significantly lower in treatment group.	[76]
RCT	52 *H. pylori* positive patients were randomized in group A (low dose mastic gum), group B (high dose mastic gum), group C (pantoprazole), and group D (standard therapy).	Pure mastic gum [350 mg (group A) or 1 g (group B) thrice daily] or combination of pure mastic gum (350 mg thrice daily) and pantoprazole (20 mg twice daily) (group C).	Standard therapy (pantoprazole 20 mg, amoxicillin 1 g and clarithromycin 500 mg) for 10 days (group D).	* H. pylori * eradication was observed in 4/13 patients in group A, 5/13 patients in group B, none of the patients in group C, and 10/13 patients in group D. No significant differences were found in mean UBT values in groups A, B and C.	[77]

^1^ Famotidine. RCT, randomized clinical trial; MDA, malondialdehyde; TAC, total antioxidant capacity; UBT, urea breath test.

**Table 2:** Summary of clinical trials of micronutrients in *H. pylori* infection.

Study Design	Study Samples	Experimental Intervention	Control Intervention	Main Outcomes	References
RCT	312 patients, with an indication for endoscopy of dyspeptic symptoms were randomized in group A (*n* = 162; mean age: 45 years) and group B (*n* = 150; mean age: 43 years).	Standard therapy (amoxicillin 1 g, metronidazole 500 mg, bismuth 240 mg and omeprazole 40 mg) plus Vitamin C 500 mg/day *(Group B)*.	Standard therapy (amoxicillin 1 g, metronidazole 500 mg, bismuth 240 mg and omeprazole 40 mg) *(Group A)*.	*H. pylori* eradication rates in group B was significantly higher (78%) than group A (48.8%).	[78]
RCT	200 patients were randomized in group A (mean age: 39.7 ± 10) and group B (mean age: 42.7 ± 10.8).	Standard triple therapy (lansoprazole, amoxicillin, and clarithromycin) for 14 days plus vitamins C (500 mg) and E (200 mg) for 30 days *(Group A)*.	Standard triple therapy alone for 14 days *(Group B)*.	*H. pylori* eradication rates in group A was significantly higher as compared to group B.	[79]
RCT	160 patients were randomized in group A (mean age: 44 ± 10) and group B (mean age: 43 ± 11)	Conventional therapy for 2 weeks plus vitamins C (1000 mg/day) and E (400 IU/day) for 1 month *(Group B)*.	Conventional anti-*H. pylori* therapy (lansoprazole, amoxicillin, clarithromycin, and bismuth subcitrate) for 2 weeks *(Group A).*	*H. pylori* eradication rates were significantly higher in group B. No difference was found in TAC among both groups.	[80]
RCT	117 patients were randomized into 4 groups: conventional therapy, vitamins (C and E), combination of both or placebo.	Triple therapy alone ( Bismuth chelate, tetracycline, and metronidazole for 2 weeks ), vitamins C (200 mg) and E (50 mg) twice a day for 4 weeks, or combination of both treatments.	Placebo	No significant effect was found on MDA levels and ROS with vitamins supplementation.	[81]
RCT	171 *H. pylori* infected patients.	One-week triple therapies of omeprazole and amoxicillin, plus on the following twice daily: (1) clarithromycin 250 mg; (2) clarithromycin 250 mg and vitamin C 500 mg; (3) clarithromycin 500 mg	Clarithromycin 250 and 500 mg.	Combination of clarithromycin 250 and vitamin C showed higher eradication rates than clarithromycin 250 mg, and equivalent eradication rates to clarithromycin 500 mg.	[82]
RCT	400 patients, with non-ulcer dyspepsia. The patients were randomized in 4 groups, 100 patients each: Group A aged 26–66 years), Group B (aged 21–65 years), Group C (aged 24–66 years) and Group D (aged 22–65 years).	Triple therapy for 2 weeks plus vitamins C (500 mg/day) and E (100 U/day) for 1 month *(Group B)*.Quadruple therapy for 2 weeks plus vitamins C (500 mg/day) and E (100 U/day) for 1 month *(Group D)*.	Standard triple therapy (amoxicillin, clarithromycin, and lansoprazole) for 2 weeks *(Group A)*. Standard quadruple therapy (amoxicillin, clarithromycin, lansoprazole, and bismuth subcitrate) for 2 weeks *(Group C)*.	No difference was found in *H. pylori* eradication rates among Groups A and B, and similarly Group C and D.	[83]
RCT	332 patients with *H. pylori* associated gastritis, aged 18–70 years infection.	Polaprezinc ^1^ 75 mg plus triple therapy twice daily for 14 days. Polaprezinc 150 mg plus triple therapy twice daily for 14 days.	Triple therapy (omeprazole, amoxicillin, and clarithromycin) for 14 days.	Triple therapy in combination with Polaprezinc 75/150 mg showed higher efficacy in eradication of infection, with no significant difference between both groups.	[84]
RCT	66 patients (mean age: 48.5 years) suffering from dyspeptic symptoms with *H. pylori* infection.	Triple therapy plus polaprezinc 150 mg twice daily.	Triple therapy (lansoprazole, amoxycillin and clarithromycin) twice daily.	Triple therapy in combination with polaprezinc showed in improvement of infection eradication rates.	[85]
RCT	90 patients with peptic ulcer disease and with mean age 47.5 ± 17.2 years in intervention group, and 52.6 ± 18.4 years in placebo group.	Standard triple therapy plus zinc 220 mg/day for 2 weeks.	Placebo	No significant difference was found between both groups in infection eradication, and improvement of peptic ulcer disease.	[86]

^1^ A chelated form of zinc and L-carnosine. RCT, randomized clinical trial; ROS, reactive oxygen species; TAC, total antioxidant capacity.

## Data Availability

Data sharing is not applicable to this article.
